# Footbathing and Foot Trimming, and No Quarantine: Risks for High Prevalence of Lameness in a Random Sample of 269 Sheep Flocks in England, 2022

**DOI:** 10.3390/ani14142066

**Published:** 2024-07-14

**Authors:** Katharine Eleanor Lewis, Martin Green, Rachel Clifton, Emma Monaghan, Naomi Prosser, Elizabeth Nabb, Laura Green

**Affiliations:** 1 School of Veterinary Medicine and Science, University of Nottingham, Nottingham NG7 2RD, UK; martin.green@nottingham.ac.uk (M.G.); rachel.clifton@nottingham.ac.uk (R.C.); naomi.prosser@nottingham.ac.uk (N.P.); 2 Institute of Microbiology and Infection, University of Birmingham, Birmingham B15 2TT, UK; e.monaghan@bham.ac.uk; 3 Animal & Plant Health Agency Veterinary Investigation Centre, Starcross, Exeter EX6 8PE, UK

**Keywords:** ewes, lambs, lameness, quarantine, footbathing, foot trimming, exploratory variable selection

## Abstract

**Simple Summary:**

Our aims were to investigate whether the Farm Animal Welfare Committee 2011 target of prevalence of lameness in English sheep flocks of <2% by 2021 had been achieved and to identify management practices associated with controlling lameness. A retrospective postal survey was sent to a random sample of 1000 English farmers. The usable response percentage was 26.9%. The geometric mean prevalence of lameness was <2% in ewes and lambs, but the median was 3%; approximately 26% flocks had <2% lameness. Farmers that quarantined ewes for 3 or more weeks and did not use footbathing or foot trimming to prevent lameness had a 40–50% lower prevalence of lameness than those not using these practices. More farmers were not using routine foot trimming than in 2018, which is a positive impact. However, more farmers were footbathing sheep, and fewer farmers were using injectable antibiotics to treat footrot, a bacterial disease, which requires antibiotic injection for recovery and to minimise spread of disease. We conclude that the target of <2% lameness in England has been achieved by 26% of farmers, and further work is required for more farmers to follow the evidence-based management practices to minimise lameness.

**Abstract:**

Since 2004, the prevalence of lameness in sheep flocks in England has reduced as farmers have adopted evidence-based management practices to control lameness. In 2011, the Farm Animal Welfare Council proposed a target prevalence of <2% lameness in sheep by 2021. This study investigated whether that target had been achieved and determined which practices were associated with prevalence of lameness. A postal questionnaire was sent to 1000 randomly selected farmers to investigate the prevalence of lameness and management practices in 2022. The geometric mean prevalence of lameness was <2% in ewes and lambs, but the median was 3%; approximately 26% flocks had <2% lameness. Data were analysed using robust variable selection with multivariable linear models. Farmers that quarantined ewes for ≥3 weeks and did not use foot bathing or foot trimming to prevent lameness had 40–50% lower prevalence of lameness than those not using these practices. Fewer farmers (19.0%) were always using parenteral antimicrobials to treat footrot, an effective practice, than in previous research (49.7%). We conclude that the target of <2% lameness in England has been achieved by 26% of farmers, and further work is required for more farmers to follow the evidence-based management practices to minimise lameness.

## 1. Introduction

Lameness is an important concern for poor health and welfare of sheep globally. In England, most lameness is caused by footrot, an infectious bacterial disease caused by *Dichelobacter nodosus.* Footrot has two clinical presentations: interdigital dermatitis (ID), an inflammation of the interdigital skin, and severe footrot (SFR), where the hoof horn separates from the underlying tissue [1,2]. Effective management practices that reduce occurrence of both infectious and non-infectious causes of lameness in ewes and lambs [3,4,5,6,7] include treatment of lame sheep within 3 days of onset of lameness and treatment of sheep with bacterial lameness with parenteral and topical antimicrobial agents [8,9], practising quarantine for new and returning sheep for ≥3 weeks and separating lame sheep for treatment, and not using flock management practices of foot trimming and footbathing [7,10].

When farmers follow recommended practices for prompt and appropriate treatment of footrot, flock prevalence of lameness is <2% [7,8,11]. This evidence led the Farm Animal and Welfare Council (FAWC), an independent advisory body to GB governments, to propose in 2011 that the prevalence of lameness in the national flock should be <2% by 2021 [12]. Since 2006, there has been considerable promotion of management practices to control lameness in sheep in England, highlighting the evidence for prompt appropriate treatment of lame sheep and, more recently, a set of practices grouped as the Five Point Plan [13,14]. The prevalence of lameness in sheep in England has reduced from a global mean period prevalence of lameness of 10.6% in 2004 [3] to 4.9% in 2013 [7] and 1.4% in 2018 [5] as more farmers use more evidence-based managements (e.g., Winter et al., 2015 [7]; Lewis et al., 2021 [5]). However, when farmers stop using evidence-based management practices to control lameness, prevalence of lameness increases [11].

Much research on lameness in sheep has been collected from postal questionnaires [3,5,7,11]. Response rates have been reasonable and farmers can reliably estimate and recall prevalence of lameness in their flock [15], so questionnaires are a reasonable method to investigate lameness in sheep. Questionnaires typically have a large number of variables relative to the number of responses, and so traditional approaches to multivariable analysis risk overfitted models with false positive variables selected [16]. This can be overcome with exploratory variable selection to determine sparse models using different combinations of predictor variables [17]. The code to analyse data using this approach is now available in the R package *HCModelSets* [18].

The aim of this study was to estimate the prevalence of lameness in sheep in England in 2022 to determine whether the FAWC target had been met and to identify which management practices were most important in controlling lameness in lambs and ewes in 2022 using robust variable selection.

## 2. Materials and Methods

Ethical approval was granted by University of Birmingham (project 2285915, approval number ERN_2022-0483).

### 2.1. Questionnaire Design and Administration

The questionnaire (Supplementary File S1) was designed by authors LG, KL, RC, EN, and NP, based on peer review publications and experience of managing lameness in sheep. The questionnaire was sent to a random sample of 1000 sheep farmers in England selected by random number from the Agriculture and Horticulture Development Board (AHDB) Better Returns member list. Administration of the questionnaire and data entry were managed by Cleardata (Cleardata UK Ltd., Northumberland, UK). The questionnaire and cover letter were sent to farmers on 6 June 2023, a postcard reminder was sent to non-responders on 20 June 2023, and the questionnaire and cover letter were sent to all non-responders on 4 July 2023. Data were entered into a specifically designed Excel template. Five questionnaires that were received after the deadline for returns of 8 August 2023 were entered onto the template manually by KL.

### 2.2. Response Rate and Data Cleaning

There were 439 (43.9%) responses; 90 farmers had no sheep, or had retired or died. There were 269 (26.9%) useable responses, which included an estimate of prevalence of lameness in ewes and lambs and flock size. Data were cleaned and analysed in R Version 4.2.2 [19]. The numbers and percentage of farmers practising each management are in Supplementary File S2: Table S1.

### 2.3. Descriptive Statistics

The farmers’ estimate of the average level of lameness in ewes and lambs was used to calculate the geometric mean and non-parametric measures and also grouped into four categories, <2%, 2–<5%, 5–<10%, and ≥10%, which farmers consider good, average, poor and unacceptable prevalence of lameness, respectively [20]. These figures were compared with those from the most recent previous survey in 2018 [5].

The percentage of farmers who reported following each point in the Five Point Plan (treat, vaccinate, cull, quarantine, avoid) was summarised and tabulated with the prevalence of lameness and internally validated using responses in the questionnaire to investigate whether farmers complied with management practices in that Point. The number and percentage of farmers practising managements in 2022 were also compared with the 2018 survey.

### 2.4. Linear Model for Associations between Management Practices and Prevalence of Lameness in Ewes and Lambs

The natural log of the prevalence of lameness in ewes and lambs were used as outcome variables to investigate management practices associated with prevalence of lameness in ewes and lambs, respectively. Flocks with no lameness were excluded from the analysis because farmers were not using many treatment practices (Supplementary File S2: Table S2). Variables for ‘ideal treatment’ and ‘ideal prevention’ were made by combining responses to several questions. Categorical predictor variables were transformed into dummy (yes/no) variables using *fastDummies* [21]; there were 68 dummy predictor variables and no continuous variables. Predictor variables were reduced to a smaller number using Cox and Battey’s 2017 method [18] for wide data, when the number of explanatory variables is large relative to the number of study participants. Briefly, to reduce the number of predictor variables and identify a small number (<8) of ‘important’ variables, variables were randomly placed into three dimensions (*p* * *p* * *p* hypercubes, where *p* is determined by taking the cube root and rounding to the nearest integer such that *p* * *p* * *p* is at least as large as the total number of variables; here, *p* is 5). Linear models were run on subsets of 5 or fewer variables from the hypercubes, and when variables were significant (*p*-value of 0.05) in ≥2 subsets, they were carried forward in the analysis. This reduction phase was repeated ten times, and variables were considered important if selected in ≥9 repeats. As part of the exploratory phase, Spearman’s rank correlation coefficients were calculated between all pairs of the subset of variables that warranted further analysis. Interactions and squared terms for continuous predictors (if they had been present) were examined among these important variables and were included if significant and biologically important. There were 5 and 4 important variables selected to model for ewes and lambs, respectively. Final ‘best’ models were constructed using the *leaps* package [22], which compares all combinations of variables to find the model that minimises the Bayesian Information Criterion (BIC). Model fit of the final models was assessed by analysis of the residuals.

Coefficients from model risk factors were exponentiated, and the proportion of lameness attributable to the risk factors was calculated.

## 3. Results

### 3.1. Flock Characteristics and Lameness Prevalence

**Figure 1:**
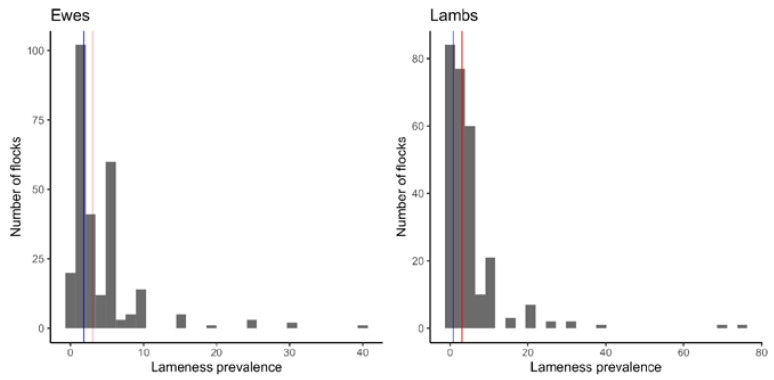
Flock prevalence of lameness in ewes and lambs in 269 flocks in England in 2022. Blue line—geometric mean prevalence (1.8% in ewes, 0.8% in lambs), red line—median prevalence (3% in both ewes and lambs).

The median flock size was 195 ewes, range 4–4000 (Table 1). Lameness ranged from 0–40% in ewes and 0–75% in lambs. There was no lameness in 13 ewe flocks and 37 lamb flocks. The distribution of lameness in ewes and lambs was right-skewed, with reporting biases around 5 and 10 percentiles (Figure 1). The geometric mean prevalence of lameness was 1.8% (95% confidence interval = 1.3–2.4%) in ewes and 0.8% (95% confidence interval = 0.5–1.2%) in lambs, and the median prevalence of lameness was 3% in both ewes and lambs. In 2018, the median prevalence of lameness was 2.4% in ewes and 3% in lambs [5].

Fewer flocks had <2% lameness and slightly more flocks had ≥10% lameness in ewes and lambs in 2022 than in 2018 (Table 2).

### 3.2. Causes of Lameness

The prevalence of each of six foot lesions varied by flock, with infectious causes of lameness more common than non-infectious causes in both ewes and lambs (Table 3). Footrot, SFR and ID combined, was the most common cause of lameness as in all previous studies of English sheep flocks [3,7].

### 3.3. Management Practices in 2022 Compared to 2018

The percentage of farmers following some recommended practices increased, e.g., not routine foot trimming increased from 67.8% in 2018 to 72.9% in 2022, vaccinating with FootVax™ increased from 26.7% in 2018 to 39.4% in 2022, and more farmers had used FootVax™ for >5 years (Table 4). However, the percentage of farmers using antibiotic injection to treat footrot (a recommended practice) decreased and the percentage of farmers using footbathing to treat footrot (known to be ineffective) increased (Table 5).

### 3.4. The Five Point Plan

There were 173/256 (67.6%) farmers who reported lameness in their flock who were aware of the Five Point Plan. The percentage of farmers following each point of the plan varied: treat (80.9%), cull (48.8%), vaccinate (26.2%), avoid (22.3%), and quarantine (27.0%). The median yearly prevalence of lameness was similar between flocks using and not using the plan (Table 6).

### 3.5. Management Practices Associated with Prevalence of Lameness in Ewes

Five ‘important’ variables were identified in the reduction phase and carried forward for modelling; these were: using memory to identify lame sheep to be culled and culling lame sheep when persistently lame, which were associated with higher prevalence of lameness, and always quarantining new sheep for ≥3 weeks, using ‘ideal’ treatment for SFR and using ‘ideal’ prevention for ID, which were associated with a lower prevalence of lameness. In the final model (Table 7), always quarantining new sheep for ≥3 weeks and using ‘ideal’ prevention for ID were associated with lower prevalence of lameness. The R^2^ for the final model was 0.09, indicating that 9% of the variation in the outcome was explained by these two predictors. Model fit was adequate (Supplementary File S2: Figure S1). None of the important variables not in the final model were highly correlated with variables in the final model and therefore no alternative models to the final model were considered.

### 3.6. Management Practices Associated with Prevalence of Lameness in Lambs

Four variables selected for modelling were routine foot trimming of >0–50% of ewes and stocking densities of >8 ewes/acre, which were associated with higher prevalence of lameness, and marking lame sheep to be culled and using ideal prevention for ID, which were associated with lower prevalence of lameness. The final model (Table 8) contained only the variable using ‘ideal’ prevention for ID, which was associated with lower prevalence of lameness. This model explained 3% of the variation in the outcome (R^2^ = 0.03), and the fit was adequate (Supplementary File S2: Figure S2). No variables that warranted further investigation were highly correlated with the single variable in the final model; therefore, no alternative models to the final model were considered.

Flocks where quarantine for ≥3 weeks or ‘ideal’ prevention for ID in ewes were practised had 0.69- and 0.63-fold, respectively, the prevalence of lameness than flocks not being managed using either practice. Where both managements were practised, the flock prevalence of lameness was 0.44-fold lower than where neither were practiced, e.g., in a flock with 5% lameness, if quarantine for ≥3 weeks was introduced, the prevalence of lameness would on average fall to 3.45%, and ‘ideal’ prevention of ID was practised to 3.15% and if both were practised to 2.2%. Similarly, the use of ‘ideal’ practice to prevent ID in lambs reduced the prevalence of lameness by 0.44-fold.

## 4. Discussion

Our results show that the prevalence of lameness in sheep flocks in England is still well below that of 2004 and 2013 and is similar to estimates from 2018, a very dry year that would have resulted in less expression of footrot [23,24]. The FAWC 2011 target of <2% lameness in the national flock was achieved in 2018 and in the current study if the geometric mean for the national flock is considered the FAWC target. However, on a flock-by-flock basis, only 25.7% of flocks had <2% lameness in 2022 (Table 2), and a small proportion of flocks had a very high prevalence of lameness. A reasonable model fit was obtained using the natural log of the prevalence of lameness as the outcome variable, which suggests that those flocks with high prevalence of lameness might have been experiencing epidemic outbreaks of lameness; these occur when CODD is introduced into a naïve flock and when outbreaks of ID occur in lambs.

For flocks with >2% lameness, and not in an outbreak situation, our results indicate that the most robust management practices that would lead to 30–50% reduction in prevalence of lameness are to introduce quarantine of new sheep for ≥3 weeks and not practise footbathing or routine foot trimming to prevent ID.

Quarantine has been associated with low prevalence of lameness in ewes [5,7,25]. Quarantine prevents introduction of new diseases such as CODD and also new strains of existing diseases, e.g., *D. nodosus,* into the flock; flocks without CODD and with fewer strains of *D. nodosus* have lower prevalence of lameness and better control from vaccination [26,27]. Many farmers do quarantine some sheep, but not all sheep are quarantined, and the length of time sheep are in quarantine varies [25]. Our new results agree with previous reports that quarantine of all sheep for at least 3 weeks is necessary to lower lameness [7]. In our study, 38.7% of farmers were currently following this practice, and adopting quarantine of all sheep would reduce prevalence of lameness by about 30%, a considerable improvement. Quarantine is important for control of many infectious diseases of sheep and has been promoted in campaigns such as ‘Stamp Out Scab’, and knowledge exchange promoting adoption of rigorous quarantine for control of lameness would be useful to increase compliance with the practice.

Preventive footbathing was used by nearly 50% of farmers in the current study despite considerable evidence that footbathing is not effective. Previous research has shown that sheep that are footbathed for any reason have higher prevalence of lameness than flocks that are never footbathed (e.g., [7]). Evidence includes: footbathing to treat ID in lambs and SFR in ewes is associated with higher prevalence of lameness [4,28], and control of lameness in ewes including avoiding routine foot trimming and footbathing leads to lower prevalence of lameness in lambs [4]. Most recently, footbathing has been associated with presence of non-infectious causes of lameness in flocks [6]. Footbathing has minimal disinfecting action on the surface/horn [29] and cannot kill bacteria deep in the foot, which occurs in SFR [2]. Farmers know footbaths are not effective, but a large proportion continue to use them [30]. This cognitive dissonance might arise because of habit or tradition [30], but the continued promotion and discussion of footbaths and which of the range of footbathing chemicals is ‘best’ in knowledge exchange materials (e.g., the Five Point Plan) re-enforces the use of footbaths.

Foot trimming causes lameness when sensitive tissue in the foot is damaged [6]. It increases the occurrence of footrot, granulomas, and deformed feet and increases recurrent lameness [7,31]. Hoof horn grows continuously; in a study of one flock, the yearly growth was 15 cm and the yearly wear was 15 cm. Hoof horn is longer when the underfoot conditions are soft and shorter when they are hard [32,33]. The majority of flocks in England are now not foot trimmed routinely, which saves considerable time [34] and prevents about 30% of lameness [11]; e.g., in the current study, only 11/269 farmers reported >5% feet bled during routine foot trimming (Supplementary File S2: Table S1).

Therapeutic foot trimming is also detrimental to recovery from footrot [4]; unfortunately, many farmers still trim hoof horn as part of the treatment for lame sheep. This is contributing to a higher prevalence of lameness, and a knowledge exchange message that might help reduce use of therapeutic foot trimming is that excess hoof horn protects a lame foot and that the horn will wear away once a sheep is weight bearing after recovery.

The questionnaire focused on managements known to be associated with lameness in sheep, so the risk factors in this study are not novel. Instead, they are currently the most important focus for recommendations to maximise reduction in the national flock; quarantine, footbathing, and foot trimming are contributing to 30–50% of lameness. Some managements that are important, and previously associated with prevalence of lameness in cross-sectional studies, were not detected in the models. This might have been because many farmers have already adopted the recommendations, e.g., stopping routine foot trimming. In addition, the robust novel variable selection technique [17], which creates sparse models and minimises the risk of overfitting, led to few risk factors being identified. It might have omitted identifying management practices relevant to a small number of farmers, e.g., prompt, individual treatment of lame sheep is ‘best practice’ [9]. However, the proportion of farmers using this ‘ideal management’, i.e., parenteral antibiotics, topical antibacterials, not foot trimming, and not footbathing to treat SFR and ID, has decreased since 2018 (Table 4) and was not a large enough proportion to be detected in the models. Responsible use of antimicrobials is being encouraged in farming to contribute to the slowing of development of antimicrobial resistance. This is very important; however, it is still responsible to use appropriate antimicrobials to treat footrot and CODD, both bacterial diseases. It is worth noting that once the incidence of lameness is low in a flock, few antimicrobial treatments are required as reported by farmers with no lameness (Supplementary File S2: Table S2).

## 5. Conclusions

We conclude that the target of <2% lameness in England has been achieved by 26% of farmers. Currently, quarantine of all new sheep for at least 3 weeks and no routine foot bathing or foot trimming to prevent ID are important to reduce prevalence of lameness in sheep flocks. If more farmers were to quarantine new sheep for at least 3 weeks and avoid footbathing to treat ID, the prevalence of lameness would fall by 30–50% and more farmers would achieve the FAWC target of <2% lame sheep.

## Figures and Tables

**Table 1:** Summary of flock size and prevalence of lameness in 269 flocks in England in 2022.

	Geometric Mean	Median	Minimum	IQ_25	IQ_75	Maximum
Flock size						
Ewes	169	195	4	69	450	4000
Lambs	252	300	3	93	700	5000
Prevalence of lameness (%)						
Ewes	1.8	3	0	2	5	40
Lambs	0.8	3	0	1	5	75

IQ = interquartile range (25th and 75th percentiles).

**Table 2:** Number and percentage of ewes and lambs by category of lameness in random samples of English flocks in 2022 and 2018.

Flock	Prevalence of Lameness (N, (%))			
	<2%	2–<5%	5–<10%	≥10%
2022, 269 flocks				
Ewes	69 (25.7)	106 (39.4)	68 (25.3)	26 (9.7)
Lambs	88 (32.7)	83 (30.9)	60 (22.3)	38 (14.1)
2018 *, 304 flocks				
Ewes	105 (34.5)	126 (41.4)	49 (16.1)	24 (7.9)
Lambs	125 (41.1)	107 (35.2)	43 (14.1)	29 (9.5)

N = number of flocks, % = percentage, * Lewis et al., (2021) [5].

**Table 3:** Number, percentage, and distribution of foot lesions in ewes and lambs in 269 flocks in England.

Foot Lesion	Flocks with Lesion (N, (%))	Prevalence of Lesion in Flocks with Lesion (%)				
		Median	Min	IQR 25	IQR 75	Max
Ewes						
Interdigital dermatitis	207 (77.0)	4	0.02	2	6.5	80
Severe footrot	207 (77.0)	3	0.01	1	5.0	90
CODD	108 (40.1)	2	0.03	1	5.0	55
Granuloma	119 (44.2)	1	0.01	1	2.0	10
Shelly hoof	149 (55.4)	3	0.50	1	6.0	80
White line abscess	71 (26.4)	1	0.10	1	2.0	71
Lambs						
Interdigital dermatitis	213 (79.2)	8	0.05	3	15.00	80
Severe footrot	114 (42.4)	2	0.01	1	5.00	60
CODD	51 (19.0)	2	0.01	1	5.00	30
Granuloma	14 (5.2)	1	0.10	1	1.75	20
Shelly hoof	43 (16.0)	1	0.50	1	3.50	10
White line abscess	18 (6.7)	1	0.10	1	2.00	50

N = number of flocks, % = percentage, CODD = contagious ovine digital dermatitis, Min = minimum, IQR = interquartile range (25th and 75th percentiles), Max = maximum.

**Table 4:** Number and percentage of farmers practising ‘best practice ’managements in 269 flocks in England in 2022 compared with the number and percentage of farmers performing each management practice in 304 flocks in England in 2018.

Management Practice	‘Ideal Management’	2022 N (%)	2018 * N (%)
Foot trim to treat footrot	Never	67 (24.9)	43 (14.1)
Footbath to treat footrot	Never	110 (40.9)	225 (74.0)
Parenteral antibiotics to treat SFR	Always	51 (19.0)	151 (49.7)
Routine foot trimming	Never	73 (27.1)	110 (36.2)
Vaccination with FootVax™	1–5 years	53 (19.7)	48 (19.1)
	>5 years	53 (19.7)	23 (7.6)
Quarantine new sheep for ≥3 weeks	Always	106 (39.4)	151 (49.7)
Cull policy for lame sheep	Lame > 0 < 3 occasions	62 (23.1)	26 (8.6)

N = number of flocks, % percentage = percentage, * data from Lewis et al., 2021 [5].

**Table 5:** The number and percentage of flocks using ‘ideal’ management practices in 256 flocks with lameness in ewes in England 2022.

		Flocks with Lame Ewes (256)		Flocks with Lame Lambs (232)	
Group Variable	Management Practice	N (%)	N (%)	N (%)	N (%)
‘Ideal’ treatment of SFR in ewes			21 (8.2)		19 (8.2)
	Treat within 1 week of onset	220 (85.9)		197 (84.9)	
	Parenteral antimicrobials	197 (77.0)		182 (78.4)	
	Foot spray 1–4 feet	218 (85.2)		202 (87.1)	
	No foot trim	61 (23.8)		52 (22.4)	
‘Ideal’ prevention of SFR in ewes			12 (4.7)		12 (5.2)
	No therapeutic foot trim	109 (42.6)		108 (46.6)	
	No routine foot trim	114 (44.5)		104 (44.8)	
	No footbath	107 (41.8)		83 (35.8)	
	FootVax™ to prevent	76 (29.7)		74 (31.9)	
‘Ideal’ treatment of ID in ewes			44 (17.2)		40 (17.2)
	Parenteral antibiotics	126 (49.2)		115 (49.6)	
	Foot spray 1–4 feet	225 (87.9)		208 (89.7)	
	No foot trim	147 (57.4)		133 (57.3)	
‘Ideal’ prevention of ID in ewes			46 (18.0)		39 (16.8)
	No routine foot trim	114 (44.5)		104 (44.8)	
	No footbath to prevent	103 (40.2)		87 (37.5)	

N = number of flocks, % = percentage, ID = interdigital dermatitis, SFR = severe footrot.

**Table 6:** Farmer-reported use of each point of the Five Point Plan and median flock lameness, 256 flocks.

	Practised Point in Five Point Plan			
	No		Yes	
Point in Five Point Plan	N (%)	Median % Lame	N (%)	Median % Lame
Treat	49 (19.1)	2.0	207 (80.9)	3.0
Vaccinate	189 (73.8)	3.0	67 (26.2)	3.0
Cull	131 (51.2)	3.0	125 (48.8)	3.0
Quarantine	187 (73.0)	3.0	69 (27.0)	3.0
Avoid	199 (77.7)	3.0	57 (22.3)	3.0

N = number of flocks, % = percentage.

**Table 7:** The final ‘best’ model for management practices associated with prevalence of lameness in 256 flocks with lame ewes in England in 2022.

Predictor		N (%)	β	Confidence Interval	
Intercept			1.29	1.15	1.43
Always quarantined new sheep for ≥3 weeks	No	157 (61.3)	Ref		
	Yes	99 (38.7)	−0.37	−0.58	−0.15
‘Ideal’ prevention for ID	No	210 (82.0)	Ref		
	Yes	46 (18.0)	−0.46	−0.73	−0.19

ID = interdigital dermatitis, β = model coefficient (natural log scale), Ref = reference category.

**Table 8:** The final model for management practices associated with prevalence of lameness in 232 lamb flocks in England in 2022.

Predictor		N (%)	β	Confidence Interval	
Intercept			1.25	1.11	1.39
‘Ideal’ prevention for ID	No	193 (83.2)	Ref		
	Yes	39 (16.8)	−0.44	−0.79	−0.10

ID = interdigital dermatitis, β = model coefficient (natural log scale), Ref = reference category.

## Data Availability

Data will not be available publicly as consent to do so was not obtained from study participants.

## Supplementary Materials

The following supporting information can be downloaded at: https://www.mdpi.com/article/10.3390/ani14142066/s1, Supplementary File S1: Questionnaire; Supplementary File S2: Table S1. Number and percentage of flocks where each management was practised in the 269 flocks. Table S2. Management practices in the 13 flocks reporting no lameness in ewes in 2022. Figure S1: Visual assessment of the ‘best’ model fit for ewes. Figure S2: Visual assessment of the ‘best’ model fit for lambs.
