# Supplementary material for: Footbathing and Foot Trimming, and No Quarantine: Risks for High Prevalence of Lameness in a Random Sample of 269 Sheep Flocks in England, 2022
Source: Animals (Basel). 2024 Jul 14;14(14):2066. doi: 10.3390/ani14142066 (PMC11273439; doi:10.3390/ani14142066)
Supplement: Supplementary file 1 [file animals-14-02066-s001.zip › Supplementary File S1 (Questionnaire).pdf]

Questionnaire on lameness in sheep 2022

Please read each question carefully and answer the question to the best of your ability: there are no right or wrong answers, just tell us what you did, over the year in 2022. We understand that sheep farming is not the same over the whole year, so please choose the answer that represents your flock over the year.

In the first section we ask you about causes of lameness you saw in your flock

In the second section we ask you how you treated and prevented footrot and scald

In the third section we ask you some information about your flock

In the fourth section we ask you about some managements you did 2022.

For each question….

There is an instruction on how to fill it in in italics: most questions ask you to circle one or more answers or to write a number.

When we have an ‘other’ section this is so that you can add any other management practices that you do.

At the end of the questionnaire you can write your email address and we will send you a summary of the results. This sheet is separated from the questionnaire to ensure your response is anonymous.

Thank you for your contributing your time completing this questionnaire.

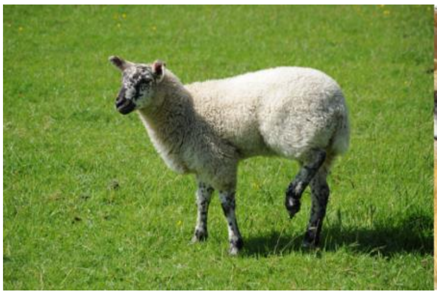
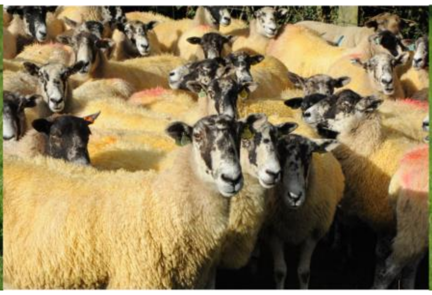
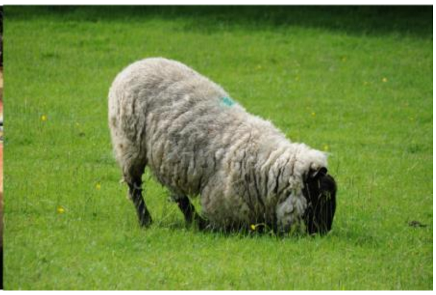

UNIVERSITY OF BIRMINGHAM

AHDB
AGRICULTURE & HORTICULTURE DEVELOPMENT BOARD

Deliberately left blank

Section 1 Causes of lameness in your flock

1. In the table below, please read the description of the lesion in the left-hand column, and look at the **example** photograph for **each cause of lameness**, and then answer the questions in the right-hand column

**Example** photograph of the lesion	**Cause of lameness** Name and description	Did this lesion cause lameness in your flock between **January 1st and December 31st, 2022**?<br>*Please answer the question below for each cause of lameness*
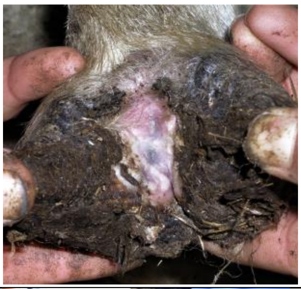	**Scald / strip**<br><br>Red, wet skin between the toes<br><br>Grey, pasty scum over skin<br><br>Loss of hair on skin between the toes	**Yes** **No** **Don't know**<br>*Please circle one answer*<br><br>*If yes*, what percentage (%) of ewes and lambs were lame with this condition?<br><br>Ewes ___________% Lambs ___________%
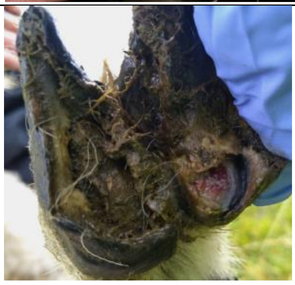	**Footrot**<br><br>Some separation of horn from the foot<br><br>Foul smelling, slimy, flesh	**Yes** **No** **Don't know**<br>*Please circle one answer*<br><br>*If yes,* what percentage of ewes and lambs were lame with this condition?<br><br>Ewes ___________% Lambs ___________%
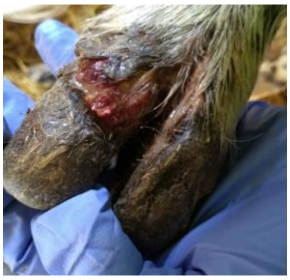	**CODD**<br><br>Blisters at hair horn junction<br><br>Red lesion prone to bleeding<br><br>May be complete detachment of hoof horn	**Yes** **No** **Don't know**<br>*Please circle one answer*<br><br>*If yes,* what percentage of ewes and lambs were lame with this condition?<br><br>Ewes ___________% Lambs ___________%
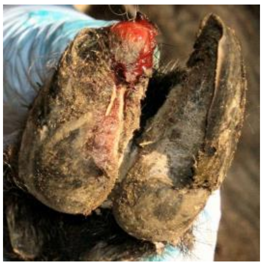	**Granuloma**<br><br>Strawberry-like growth coming out of the horn<br><br>Bleeds when handled<br><br>Very painful	**Yes** **No** **Don't know**<br>*Please circle one answer*<br><br>*If yes,* what percentage of ewes and lambs were lame with this condition?<br><br>Ewes ___________% Lambs ___________%
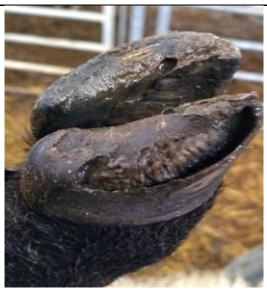	**Shelly Hoof**<br><br>Pockets of separation of hoof from foot<br><br>Can become impacted with soil and stones	**Yes** **No** **Don't know**<br>*Please circle one answer*<br><br>*If yes,* what percentage of ewes and lambs were lame with this condition?<br><br>Ewes ___________% Lambs ___________%
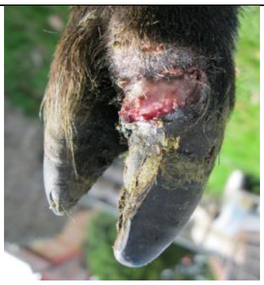	**White Line Abscess**<br><br>Foot hot and painful<br><br>No outward signs of disease<br><br>Pus coming from under hoof horn	**Yes** **No** **Don't know**<br>*Please circle one answer*<br><br>*If yes,* what percentage of ewes and lambs were lame with this condition?<br><br>Ewes ___________% Lambs ___________%

Section 2 Management of lameness

In this section we ask you about how you treat sheep with footrot and then how you treat sheep with scald / strip, then we ask you how you prevent footrot and scald in your flock

2. Between **January 1st and December 31st, 2022** which of the following did you use to **treat** ewes with **footrot**? *Please circle one answer for each **treatment***

Treatment				
Foot trimming	**Never**	**Sometimes**	**Usually**	**Always**
Foot bathing	**Never**	**Sometimes**	**Usually**	**Always**
Antibiotic injection	**Never**	**Sometimes**	**Usually**	**Always**
Foot spray affected feet	**Never**	**Sometimes**	**Usually**	**Always**
Foot spray all feet	**Never**	**Sometimes**	**Usually**	**Always**
Pain killer	**Never**	**Sometimes**	**Usually**	**Always**
Vaccination with Footvax	**Never**	**Sometimes**	**Usually**	**Always**
Separate lame sheep	**Never**	**Sometimes**	**Usually**	**Always**
Other treatment *please describe* ______________________		**Sometimes**	**Usually**	**Always**

3. Between **January 1st and December 31st, 2022** which of the following did you use to **treat** ewes with **scald / strip**? *Please circle one answer for each **treatment***

Treatment				
Foot trimming	**Never**	**Sometimes**	**Usually**	**Always**
Foot bathing	**Never**	**Sometimes**	**Usually**	**Always**
Antibiotic injection	**Never**	**Sometimes**	**Usually**	**Always**
Foot spray affected feet	**Never**	**Sometimes**	**Usually**	**Always**
Foot spray all feet	**Never**	**Sometimes**	**Usually**	**Always**
Pain killer	**Never**	**Sometimes**	**Usually**	**Always**
Vaccination with Footvax	**Never**	**Sometimes**	**Usually**	**Always**
Separate lame sheep	**Never**	**Sometimes**	**Usually**	**Always**
Other treatment *please describe* ______________________		**Sometimes**	**Usually**	**Always**

4. Between **January 1st and December 31st, 2022**, on average how soon did you treat lame sheep **after** you saw that they were lame? *Please circle one*

**Immediately**	**1-2 days**	**3-7 days**	**8-14 days**	**After 14 days**	**Did not treat any lame sheep**

5. Approximately what percentage of lame sheep did you trim during treatment _______**%** *or* **Did not trim feet**

6. Approximately what percentage of lame sheep feet bled when trimmed? __________**%** *or* **Did not trim feet**

Section 2 Management of lameness

7. Which of the following managements did you use to **prevent footrot** in your flock?
*Please circle one answer for each **prevention***

Prevention

Foot trimming	**Never**	**Sometimes**	**Usually**	**Always**
Foot bathing	**Never**	**Sometimes**	**Usually**	**Always**
Separate lame sheep	**Never**	**Sometimes**	**Usually**	**Always**
Vaccination with Footvax	**Never**	**Sometimes**	**Usually**	**Always**
Move to new pasture	**Never**	**Sometimes**	**Usually**	**Always**
Other prevention *please describe*		**Sometimes**	**Usually**	**Always**

____

8. Which of the following managements did you use to **prevent scald / strip** in your flock?
*Please circle one answer for each **prevention***

Prevention

Routine foot trimming	**Never**	**Sometimes**	**Usually**	**Always**
Foot bathing	**Never**	**Sometimes**	**Usually**	**Always**
Separate lame sheep	**Never**	**Sometimes**	**Usually**	**Always**
Vaccination with Footvax	**Never**	**Sometimes**	**Usually**	**Always**
Move to new pasture	**Never**	**Sometimes**	**Usually**	**Always**
Other prevention *Please describe*		**Sometimes**	**Usually**	**Always**

____

9. Approximately what percentage of your flock did you trim at a routine foot trim_______**%** *or* **Did not trim feet**

10. Approximately what percentage of trimmed sheep feet bled when trimmed? _________**%** *or* **Did not trim feet**

11. When did you **first** use Footvax to vaccinate sheep against footrot? *Please circle one*

**Never used**<br>*Please go to question 15*	**Within the last year**	**1-2 years ago**	**3-5 years ago**	**More than 5 years ago**

12. Have you used Footvax every year since you started using it? *Please circle one*

No **Yes** **Don't know**

13. How often did you use Footvax between **January 1st and December 31st, 2022**? *Please circle one*

Never **Once a year** **Twice a year** **Other** ______________________________ *please state*

14. Which sheep did you vaccinate with Footvax between **January 1st and December 31st, 2022**?
Please circle all that apply

**None**	**Ewes**	**Rams**	**Sheep with footrot**	**Newly purchased sheep**	**Other** ________________________<br>*Please state*

Section 3 About your flock

15. Between **January 1st and December 31st, 2022**

Approximately how many **ewes** did you have in your flock? _________

Approximately how many **ewes** were culled because of lameness? _________

Approximately how many **lambs** were born? _________

16. Between **January 1st and December 31st, 2022**

What was the average level of lameness in **ewes** in your flock? _________%

What was the average level of lameness in **lambs** in your flock? _________%

What was the highest level of lameness in **ewes** in your flock? _________%

What was the highest level of lameness in **lambs** in your flock? _________%

17. Between **January 1st and December 31st, 2022**, how did you record the identity of lame sheep?
Please circle all that apply

**Electronic hand held device**	**Mobile phone**	**Mark on sheep**	**I remember lame sheep**	**Computer**	**Paper record**	**Ear notch**	**Did not record lame sheep**	**Other**

18. Which of the statements below best describes your policy for culling ewes that had been lame between **January 1st and December 31st, 2022**? *Please circle one*

No policy **After 1 bout of lameness** **After 2 bouts of lameness** **After 3 bouts of lameness** **When persistently lame** **Other** *please specify* ________________________________

19. Were replacement ewes selected from mothers that were never lame?
Please circle one

Yes **No** **Don't know** **Did not use homebred replacement**

20. On **1st January, 2022** were you aware of the five point plan for lameness?
Please circle one

Yes **No** **Don't know**

21. Which of the five points of the plan did you use between **January 1st and December 31st, 2022**?
Please circle all that apply

Vaccinate **Cull** **Avoid** **Treat** **Quarantine** **None of these** **Not sure**

22. Were you a member of a flock health club between **January 1st and December 31st 2022**?
Please circle one

Yes **No** **Not sure**

23. Did you change any managements for lameness in **2022** from those you did in **2021**?

Yes If yes, please state changes below **No**

Section 4 Managing your flock

24. Between **January 1st and December 31st, 2022**, how many people looked after your flock?
Please circle one answer

One **Two** **More than two** *if more than 2, please state how many*____________

25. Between **January 1st and December 31st 2022**, which of the following land did you use to graze your flock?
Please circle all that apply

Owned **Rented** **Away at keep** **Shared grazing** **Other** *please state*______

26. Which of the following describes how you replace your breeding ewes?
Please circle all that apply

Buy ewes **Buy rams** **Buy lambs** **Buy semen** **Other** **Not applicable**

27. Between **January 1st and December 31st 2022,** approximately how many sheep did you buy?
Please state how many

Ewes____________ **Rams**_______________ **Lambs**_____________

28. Between **January 1st and December 31st 2022**, did you quarantine sheep arriving on your farm **for at least 3 weeks**? *Please circle one*

Never **Sometimes** **Usually** **Always** **Not applicable**

29. Did you house your flock between **January 1st and December 31st 2022**? *Please circle one*

No **Yes**, *if yes,* for how many weeks_____________________ weeks

30. What stocking rate did you typically use when grazing between **January 1st and December 31st, 2022**?
Please circle one

Less than 4 ewes per acre **4 - 8 ewes per acre** **More than 8 ewes per acre**

31. Approximately how many days in total did you spend managing lameness over the year between **January 1st and December 31st 2022**? _________________________________ days

32. Approximately how long did each of the following **flock** managements take to do, and how often did you do them to manage lameness between **January 1st and December 31st 2022**?

Activity	*Time taken*	*Number of times done over the year*	
Gather the **flock**	_________ hours	__________ times per year	not done
Foot trim the **flock**	_________ hours	__________ times per year	not done
Foot bath the **flock**	_________ hours	__________ times per year	not done
Footvax the **flock**	_________ hours	__________ times per year	not done

33. Approximately how long did each of the following **individual sheep** managements take to do, and how often did you do them to manage lameness between **January 1st and December 31st 2022**

Catch **one individual sheep** to treat lameness	________ minutes	_____________ times per year	not done
Treat **one individual lame sheep**	________ minutes	_____________ times per year	not done
Foot trim **one individual lame sheep**	________ minutes	_____________ times per year	not done
**Separate a lame sheep** from the flock	________ minutes	_____________ times per year	not done

Deliberately left blank

THANK YOU!

This is the end of the questionnaire, if you have any further comments, please put these in the box below

If you would you like to receive a summary of the results from this questionnaire, please provide your email address below

---

THANK YOU FOR COMPLETING THE QUESTIONNAIRE

PLEASE RETURN YOUR COMPLETED QUESTIONNAIRE IN THE STAMPED ENVELOPE PROVIDED
