# Supplementary material for: Footbathing and Foot Trimming, and No Quarantine: Risks for High Prevalence of Lameness in a Random Sample of 269 Sheep Flocks in England, 2022
Source: Animals (Basel). 2024 Jul 14;14(14):2066. doi: 10.3390/ani14142066 (PMC11273439; doi:10.3390/ani14142066)

Supplementary Material

Supplementary Table S1. Number and percentage of flocks where each management was practised in the 269 flocks.

**Management practice**		**N**
**Treatment of SFR**		
Foot trim to treat SFR	Never	67 (24.9)
	Sometimes	102 (37.9)
	Usually	53 (19.7)
	Always	47 (17.5)
Footbath to treat SFR	Never	110 (40.9)
	Sometimes	67 (24.9)
	Usually	49 (18.2)
	Always	43 (16)
Antibiotic injection to treat SFR	Always	51 (19)
	Usually	58 (21.6)
	Sometimes	91 (33.8)
	Never	69 (25.7)
Foot spray affected feet to treat SFR	Always	131 (48.7)
	Usually	59 (21.9)
	Sometimes	33 (12.3)
	Never	46 (17.1)
Foot spray all feet to treat SFR	Always	32 (11.9)
	Usually	37 (13.8)
	Sometimes	79 (29.4)
	Never	121 (45)
Painkiller to treat SFR	Always	15 (5.6)
	Usually	27 (10)
	Sometimes	89 (33.1)
	Never	138 (51.3)
Vaccinate with FootVax™ to treat SFR	Always	43 (16.0)

Management practice		N
	Usually	10 (3.7)
	Sometimes	18 (6.7)
	Never	198 (73.6)
Separate lame sheep to treat SFR	Always	13 (4.8)
	Usually	29 (10.8)
	Sometimes	92 (34.2)
	Never	135 (50.2)
**Treatment of ID**		
Foot trim to treat ID	Never	157 (58.4)
	Sometimes	79 (29.4)
	Usually	14 (5.2)
	Always	19 (7.1)
Footbath to treat ID	Never	116 (43.1)
	Sometimes	46 (17.1)
	Usually	51 (19.0)
	Always	56 (20.8)
Antibiotic injection to treat ID	Always	14 (5.2)
	Usually	22 (8.2)
	Sometimes	92 (34.2)
	Never	141 (52.4)
Foot spray affected feet to treat ID	Always	133 (49.4)
	Usually	47 (17.5)
	Sometimes	49 (18.2)
	Never	40 (14.9)
Foot spray all feet to treat ID	Always	44 (16.4)
	Usually	42 (15.6)
	Sometimes	82 (30.5)
	Never	101 (37.5)
Painkiller to treat ID	Always	8 (3.0)

Management practice		N
	Usually	4 (1.5)
	Sometimes	69 (25.7)
	Never	188 (69.9)
Vaccinate with FootVax™ to treat ID	Always	28 (10.4)
	Usually	8 (3)
	Sometimes	12 (4.5)
	Never	221 (82.2)
Separate lame sheep to treat ID	Always	7 (2.6)
	Usually	15 (5.6)
	Sometimes	67 (24.9)
	Never	180 (66.9)
Time to treatment of lame sheep	Immediately	31 (11.5)
	1-2 days	99 (36.8)
	3-7 days	100 (37.2)
	8-14 days	21 (7.8)
	after 14 days	3 (1.1)
	Did not treat	1 (0.4)
	Missing	14 (5.2)
**Prevention of SFR**		
Routine foot trim to prevent SFR	Never	128 (47.6)
	Sometimes	76 (28.3)
	Usually	30 (11.2)
	Always	35 (13)
Footbath to prevent SFR	Never	109 (40.5)
	Sometimes	62 (23)
	Usually	48 (17.8)
	Always	50 (18.6)
Separate lame sheep to prevent SFR	Always	14 (5.2)
	Usually	31 (11.5)

Management practice		N
	Sometimes	92 (34.2)
	Never	132 (49.1)
Vaccinate with FootVax™ to prevent SFR	Always	48 (17.8)
	Usually	13 (4.8)
	Sometimes	16 (5.9)
	Never	192 (71.4)
Move sheep to new pasture to prevent SFR	Always	13 (4.8)
	Usually	37 (13.8)
	Sometimes	107 (39.8)
	Never	112 (41.6)
**Prevention of ID**		
Routine foot trim to prevent ID	Never	197 (73.2)
	Sometimes	37 (13.8)
	Usually	17 (6.3)
	Always	18 (6.7)
Footbath to prevent ID	Never	113 (42)
	Sometimes	46 (17.1)
	Usually	53 (19.7)
	Always	57 (21.2)
Separate lame sheep to prevent ID	Always	9 (3.3)
	Usually	16 (5.9)
	Sometimes	74 (27.5)
	Never	170 (63.2)
Vaccinate sheep with FootVax™ to prevent ID	Always	37 (13.8)
	Usually	11 (4.1)
	Sometimes	11 (4.1)
	Never	210 (78.1)
Move sheep to new pasture to prevent ID	Always	10 (3.7)
	Usually	30 (11.2)

Management practice		N
	Sometimes	104 (38.7)
	Never	125 (46.5)
**Ideal managements**		
Ideal treatment for SFR	No	247 (91.8)
	Yes	22 (8.2)
Ideal treatment for ID	No	236 (87.7)
	Yes	33 (12.3)
Ideal prevention for SFR	No	256 (95.2)
	Yes	13 (4.8)
Ideal prevention for ID	No	222 (82.5)
	Yes	47 (17.5)
**Routine foot trimming**		
Percentage flock foot trimmed at routine trim	Did not trim	118 (43.9)
	>0-50	91 (33.8)
	>50	44 (16.4)
	Missing	16 (5.9)
Percentage of sheep that bled at routine foot trim	Did not trim	73 (27.1)
	0	95 (35.3)
	>0-2	43 (16)
	>2-5	19 (7.1)
	>5	11 (4.1)
	Missing	28 (10.4)
**Vaccination with FootVax™**		
Length of time FootVax™ used	Never used	154 (57.2)
	Within last year	4 (1.5)
	1-2 years	14 (5.2)
	3-5 years	35 (13)
	>5 years	53 (19.7)
	Missing	9 (3.3)

Management practice		N
FootVax™ used every year since started using	Never used	122 (45.4)
	No	78 (29)
	Unknown	1 (0.4)
	Yes	57 (21.2)
	Missing	11 (4.1)
Frequency of use of FootVax™	Never used	192 (71.4)
	Once a year	51 (19)
	Twice a year	10 (3.7)
	Other	2 (0.7)
	Missing	14 (5.2)
Did not vaccinate any sheep with FootVax™	No	197 (73.2)
	Yes	72 (26.8)
Ewes vaccinated with FootVax™	No	209 (77.7)
	Yes	60 (22.3)
Rams vaccinated with FootVax™	No	216 (80.3)
	Yes	53 (19.7)
Sheep with footrot vaccinated with FootVax™	No	262 (97.4)
	Yes	7 (2.6)
Newly purchased sheep vaccinated with FootVax™	No	244 (90.7)
	Yes	25 (9.3)
**Recognition and marking of lame sheep**		
Used electronic device	No	246 (91.4)
	Yes	23 (8.6)
Used mobile phone	No	233 (86.6)
	Yes	36 (13.4)
Marked sheep	No	66 (24.5)
	Yes	203 (75.5)

Management practice		N
Remember sheep	No	214 (79.6)
	Yes	55 (20.4)
Used computer	No	245 (91.1)
	Yes	24 (8.9)
Used paper record	No	179 (66.5)
	Yes	90 (33.5)
Used ear notch	No	259 (96.3)
	Yes	10 (3.7)
Did not record lame sheep	No	241 (89.6)
	Yes	28 (10.4)
**Culling and replacements**		
Culling policy for lame sheep	No policy	61 (22.7)
	after 1	11 (4.1)
	after 2	51 (19)
	after 3	38 (14.1)
	When persistently lame	93 (34.6)
	Missing	15 (5.6)
Replacement ewes bred from mothers that were never lame	Unknown	64 (23.8)
	Did not breed replacements	54 (20.1)
	No	91 (33.8)
	Yes	60 (22.3)
**Flock details**		
Member of a flock health club	No	217 (80.7)
	Not sure	10 (3.7)
	Yes	42 (15.6)
Managements for lameness changed in 2022 from 2021	No	197 (73.2)
	Yes	46 (17.1)
	Missing	26 (9.7)

Management practice		N
Number of people looking after flock	One	97 (36.1)
	Two	136 (50.6)
	More than two	31 (11.5)
	Missing	5 (1.9)
Land type—owned	No	54 (20.1)
	Yes	215 (79.9)
Land type—rented	No	94 (34.9)
	Yes	175 (65.1)
Land type—at keep	No	224 (83.3)
	Yes	45 (16.7)
**Purchase of new sheep**		
Ewes bought	No	150 (55.8)
	Yes	119 (44.2)
	Missing	0 (0)
Rams bought	No	89 (33.1)
	Yes	179 (66.5)
	Missing	1 (0.4)
Lambs bought	No	237 (88.1)
	Yes	32 (11.9)
	Missing	0 (0)
Semen bought	No	267 (99.3)
	Yes	2 (0.7)
	Missing	0 (0)
Quarantine of new sheep for at least 3 weeks	Always	106 (39.4)
	Usually	51 (19)
	Sometimes	27 (10)
	Never	39 (14.5)
	Missing	46 (17.1)
Flock housed	No	93 (34.6)

Management practice		N
	Yes	144 (53.5)
	Missing	32 (11.9)
Stocking density	<4 ewes/acre	101 (37.5)
	4-8 ewes/acre	24 (8.9)
	>8 ewes/acre	139 (51.7)
	Missing	5 (1.9)

1. N = number of flocks, SFR = severe footrot, ID = interdigital dermatitis

Supplementary Table S2. Management practices in the 13 flocks reporting no lameness in ewes in 2022.

Management practice	Frequency of use	N (%) flocks practising managemen t
Parenteral antibiotics to treat SFR	No	8 (61.5)
	Yes	5 (38.5)
Foot trimming to treat SFR	No	6 (46.2)
	Yes	7 (53.8)
Footbathing to treat SFR	No	9 (69.2)
	Yes	4 (30.8)
Quarantine new sheep for ≥3 weeks	Never	1 (7.7)
	Sometimes	1 (7.7)
	Usually	1 (7.7)
	Always	7 (53.8)
	Missing	3 (23.1)
Routine foot trim flock	Did not trim	3 (23.1)
	Trimmed without bleeding	6 (46.2)
	Missing	4 (30.8)
Vaccination	Never used	9 (69.2)
	>5 years	3 (23.1)

Management practice	Frequency of use	N (%) flocks practising managemen t
	Missing	1 (7.7)

1. N = number of flocks, % = percentage

Supplementary Figure S1: Visual assessment of the ‘best’ model fit for ewes.

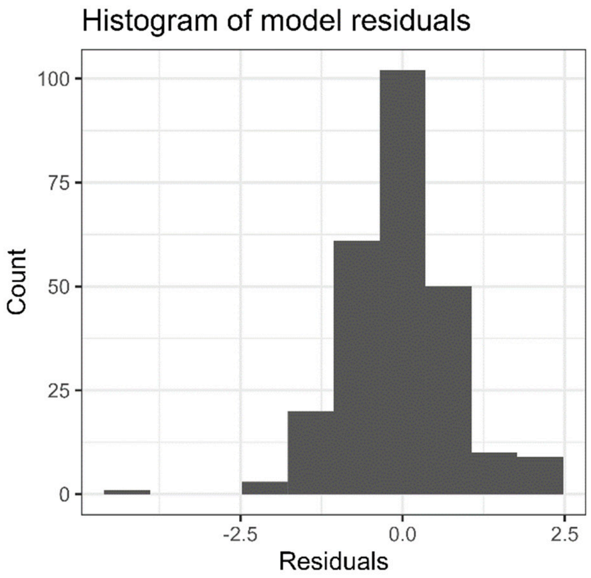

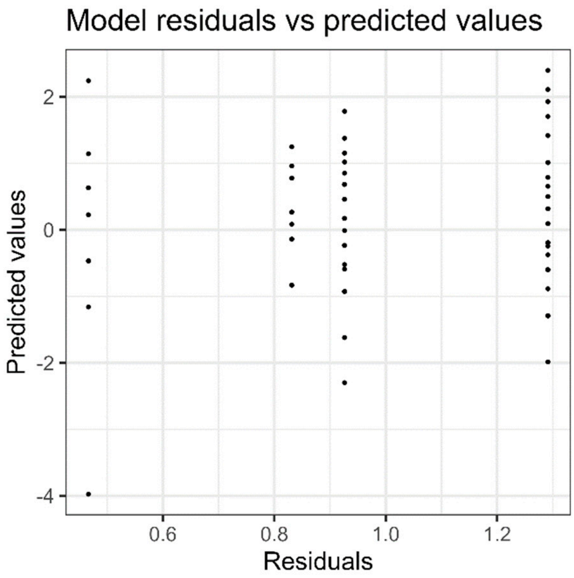

Supplementary Figure S2: Visual assessment of the ‘best’ model fit for lambs.

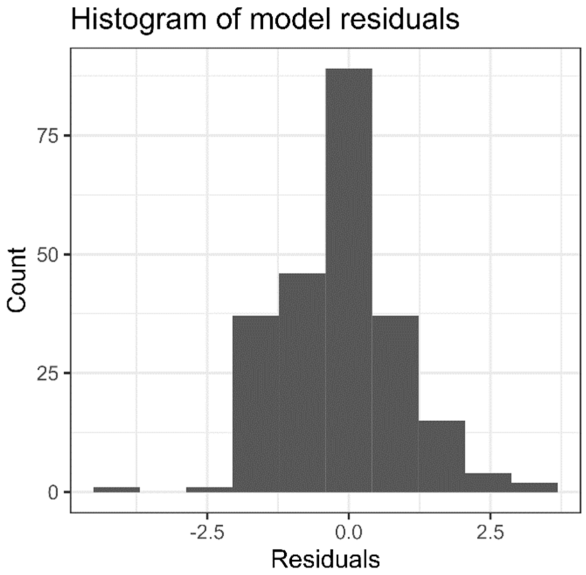

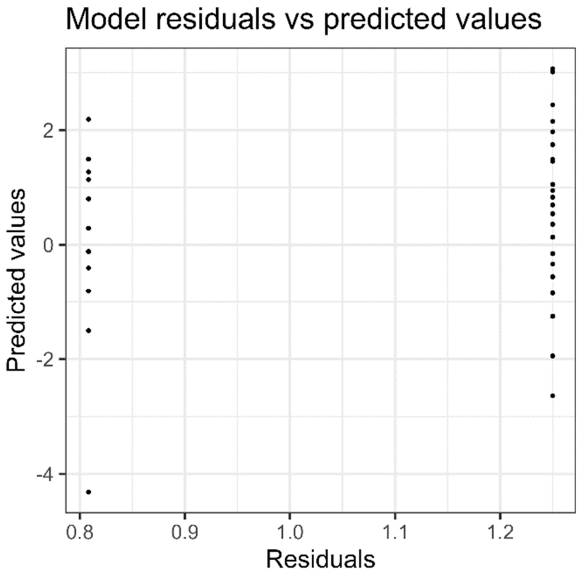

Supplement: Supplementary file 1 [file animals-14-02066-s001.zip › Supplementary File S2.pdf]
